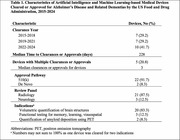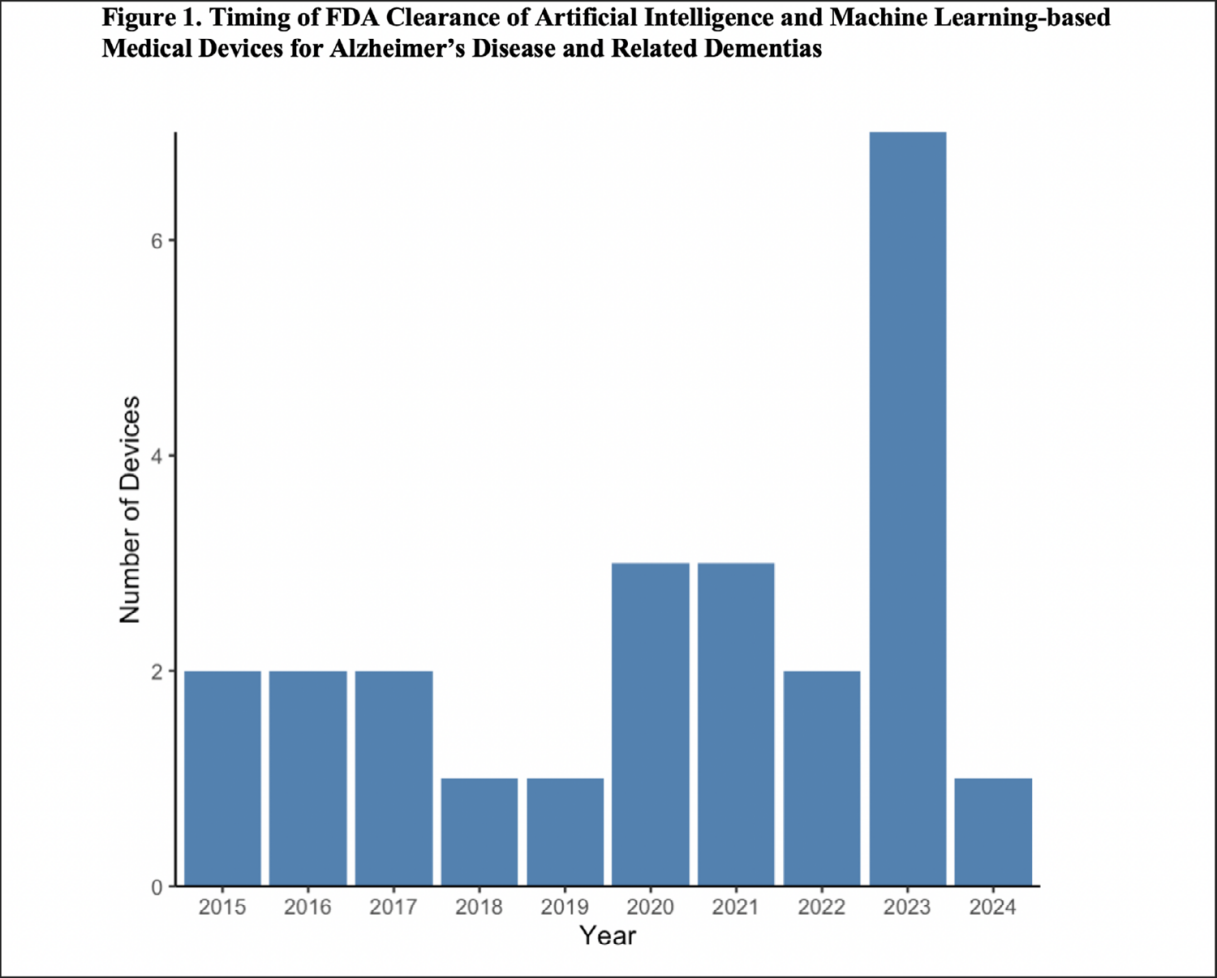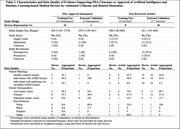# Artificial Intelligence in Diagnosis and Management of Alzheimer's Disease and Related Dementias: Evaluation of FDA Device Regulation

**DOI:** 10.1002/alz70860_102278

**Published:** 2025-12-23

**Authors:** Krista Y. Chen, Joseph S. Ross, Adam B. Cohen, Jason Karlawish, Esther S. Oh, Ravi Gupta

**Affiliations:** ^1^ Johns Hopkins University School of Medicine, Baltimore, MD, USA; ^2^ Yale School of Medicine, New Haven, CT, USA; ^3^ University of Pennsylvania Perelman School of Medicine, Philadelphia, PA, USA

## Abstract

**Background:**

Timely, accurate detection of Alzheimer's Disease and Related Dementias (ADRD) is essential for care, including treatment with disease‐modifying therapeutics. Artificial intelligence and machine learning (AI/ML)‐based medical devices have shown promise for improving diagnosis and care. However, concerns about their accuracy and potential to exacerbate health disparities underscore the need for transparency in their development and approval by the Food and Drug Administration (FDA).

**Method:**

We identified FDA‐cleared AI/ML‐based devices for ADRD since 2015 using the FDA AI/ML‐Enabled Medical Devices list, FDA 510(k) and De Novo Databases, and Google searches, as of December 2024. For identified devices, we reviewed FDA approval summary documents and peer‐reviewed literature. For devices with multiple FDA clearances, only the first was considered. Data were extracted on the regulatory approval process, indications for use, and availability and quality of training or validation datasets (i.e., study design, sample size, and demographic factors). Descriptive analyses were performed for devices with available data.

**Result:**

We identified 24 devices cleared or approved by the FDA for ADRD since 2015 (Table 1), half between 2021‐2024 (Figure 1). Most devices were cleared via the 510(k) pathway (91.7%), while few were approved under De Novo classification (8.3%). Indications included volumetric quantification of brain structures (79.2%), functional testing of memory, learning, visuospatial awareness (12.5%), and quantification of amyloid deposition (8.3%). FDA summaries reported training and validation data for 10 (41.7%) and 2 (8.3%) devices, respectively, while peer‐reviewed articles reported data for 8 (33.3%) and 10 (41.7%) devices, respectively (Table 2). Devices were primarily trained and validated on retrospective data from multicenter studies, with mean sample sizes between 261‐845 patients. Patient pathology, age, and sex distribution were reported in most peer‐reviewed articles but less than half of FDA summaries. Race/ethnicity distribution was available for at most one device from each reporting source.

**Conclusion:**

We found that the availability of evidence supporting FDA‐cleared AI/ML‐based devices for ADRD, including training and validation datasets and demographic characteristics of patients involved in algorithm development, was limited. These gaps limit clinicians’ ability to make informed decisions when using these devices, raising concerns of unintended outcomes for patients living with ADRD, particularly racial/ethnic minorities.